# The importance of the socio-historical context: similarities and differences in identity development and psychological adjustment across two cohorts of Spanish college emerging adults

**DOI:** 10.3389/fpsyg.2026.1739488

**Published:** 2026-05-28

**Authors:** Paula Domínguez-Alarcón, Inmaculada Sánchez-Queija, Águeda Parra

**Affiliations:** Department of Developmental and Educational Psychology, Universidad de Sevilla, Sevilla, Spain

**Keywords:** emerging adulthood, gender differences, identity, mental health, wellbeing

## Abstract

Identity is a psychosocial construct shaped by the generational context, reflecting culturally and historically specific patterns of self-development. This study explores and compares the identity development and psychological adjustment of two generations of emerging adults. The total sample comprised 2,819 college students (38% men, 62% women) from two cohorts surveyed: Ch1 (2015), of 1,301 emerging adults, 40.9% men, 59.1% women; *M_age_* = 20.06, *SD* = 2.03 and Ch2 (2020, which coincides with the COVID-19 pandemic), of 1,518 participants, 35.6% men, 64.4% women; *M_age_* = 20.48, *SD* = 2.48. The two cohorts show distinct patterns in *commitment making*, *identification with commitment*, and *ruminative exploration*. Ch2 exhibited lower levels on both commitment dimensions, alongside heightened *ruminative exploration*. Concurrently, Ch2 reported reduced wellbeing and increased psychological distress relative to Ch1. Moreover, the associations between identity dimensions and indicators of psychological adjustment were especially pronounced among men and within Ch2. The findings highlight how the interplay of identity development and psychosocial adjustment works in different time periods. It also underlines the need for gender perspective-focused research and intervention programs designed to foster identity consolidation during emerging adulthood during crisis times.

## Introduction

### Identity development and psychological adjustment during emerging adulthood

Identity has been defined as a process of psychosocial development that enables individuals to integrate traits that characterize and differentiate them from other individuals or groups (Erikson, 1968; Marcia, 1966, 1980). Several identity development models have been developed in the last few decades based on Erikson and Marcia’s formulations, and most have two identity concepts in common: commitment and exploration (Schwartz et al., 2013). Exploration refers to the process of gathering information about various potential commitments, such as friendship or romantic relationships, career paths, jobs, residences, family formation, or parenthood. Commitment is the degree of personal investment that an individual exhibits in their choices. The *Dual-Cycle Model*, developed by Luyckx et al. (2005), divides the commitment and exploration dimensions into subdimensions to expand Erikson and Marcia’s theory and have a better understanding of the identity development processes. The model divides the commitment dimensions into *commitment making*, equivalent to the “commitment” dimension of Erikson and Marcia, that reflect the stage where individuals settle on a choice, and the *identification with commitment*, which refers to the degree of identification of the person’s values, beliefs, and aims with the chosen commitment. Exploration dimensions are also divided into *exploration in breadth*, or the exploration and gathering of information of potential commitments (equivalent to the “exploration” dimension of Erikson and Marcia), *exploration in depth*, or the gathering of information about the chosen commitments to evaluate the degree of personal alignment with said commitment (in fact, leads to the evaluation of the identification of commitment), and *ruminative exploration,* or a perfectionist and obsessive type of exploration characterized by chronic doubt that leads to difficulty making and keeping to commitments (Luyckx et al., 2008a). The model identifies two interrelated cycles (Luyckx et al., 2006a): the commitment formation cycle (exploration in breadth-commitment making) and the commitment assessment cycle (exploration in depth-identification with commitment). Both enable the gradual consolidation of adult identity.

Identity formation was initially posited as a developmental milestone associated with adolescence (Erikson, 1968). However, in response to the sociocultural and economic transformations of the 21st century, a new developmental stage has been identified between the ages of 18 and 29, known as *emerging adulthood* (Arnett, 2000, 2014). Identity exploration and consolidation represent core tasks during this stage. This prolonged period of psychosocial exploration is made possible by the postponement of adult role adoption and the relatively limited obligations and responsibilities typically encountered during these years. These conditions enable emerging adults to engage in in-depth identity work through processes of exploration and commitment (Côte and Schwartz, 2002; Luyckx et al., 2008c). Such conditions foster the gradual maturation and consolidation of identity during emerging adulthood (Waterman, 1982; Waterman and Archer, 1990).

The identity development of emerging adults is a necessary process for youths to achieve a clearer sense of purpose and direction in life, but research has highlighted the great challenge it represents (Lillard, 2023). Moreover, previous research has demonstrated a significant association between distinct identity dimensions and indicators of psychological adjustment across both adolescence and emerging adulthood (Brinthaupt and Scheier, 2022; Schwartz et al., 2015). Specifically, *commitment dimensions* (commitment making and identification with commitment) are positively associated with higher self-esteem and wellbeing levels in emerging adults (Luyckx et al., 2013b), and negatively associated with mental health problems such as depression, anxiety, and stress (Crocetti et al., 2009; Sánchez‐Queija et al., 2023; Schwartz et al., 2012, 2015). The opposite pattern has been found for ruminative exploration, which is considered the maladaptive component of identity: it is positively associated with distress, self-rumination, and weak commitments (Beyers and Luyckx, 2016; Luyckx et al., 2008a, 2008b, 2008c), and negatively associated with self-esteem (Redmayne, 2017). Nevertheless, the evidence regarding the relationship between the exploration in breadth and exploration in depth dimensions and psychological adjustment remains less consistent. Some studies have reported negative associations between exploration in breadth and in depth, and symptoms of depression, anxiety, and stress, alongside positive associations with wellbeing (Baggio et al., 2017; Sánchez‐Queija et al., 2023). In contrast, other authors have found negative associations of the three exploration dimensions with self-esteem (Luyckx et al., 2013b), and positive associations with depression, anxiety, and stress (Luyckx et al., 2006a, 2006b). This might reflect how those exploration dimensions, which could be positive for the development of certain populations of emerging adults (except ruminative exploration), may also be negative for the psychosocial development of other collectives of emerging adults. Multiple reasons can be behind the apparent ambivalence of the exploration dimensions: whether the youths are in their early or late years of the emerging adulthood (Luyckx et al., 2013a, 2013b), the socioeconomic circumstances (Skhirtladze et al., 2016), or the time period (Cordingley, 2022). Such possibilities reflect how complex the interplay of personal circumstances with socioeconomic scenarios can be in the process of identity development. Moreover, these findings underscore the significant association of identity development with the long-term psychological functioning of emerging adults. Given the influence of the sociocultural context on identity trajectories (Galliher et al., 2017), it becomes particularly relevant to examine how these processes unfold across different generations of emerging adults. Such an approach can deepen our understanding of how identity construction and its association with psychosocial adjustment are shaped by contextual variables.

### COVID-19 pandemic

The SARS-CoV2 pandemic, also known as the COVID-19 Pandemic, was a sociohistorical turning point for humanity. It was declared a *Public Health Emergency of International Concern* by the World Health Organization (WHO) at the start of 2020, and a *Global Pandemic* only 2 months after, on March 11 of the same year (Hiscott et al., 2020). Responses to the COVID-19 pandemic included social distancing, the use of facial masks, policies of non-essential business closure, and lockdowns at home (Song et al., 2021). These measures significantly affected the lives of all citizens around the globe far beyond mere physical health; psychological and social health were also affected in several ways (Dzúrová and Květoň, 2021; Shanahan et al., 2022). Most recent studies revealed that COVID-19 restrictions had, indeed, a negative impact on mental health (Díez et al., 2024; Torales et al., 2020). In fact, this event profoundly impacted the psychosocial development of tertiary education students, many of whom were emerging adults (Daniel, 2020). The emerging adulthood was formulated as a self-centered stage focused on identity exploration and development (Arnett, 2014); that is, emerging adults could focus on the exploration of several life areas and options (i.e., friendships, partners, careers/degrees, residence options) thanks to the few limitations they had (less responsibilities than a regular adult, more freedom than a regular teen) (Côte and Schwartz, 2002). This identity exploration is key to attaining identity achievement, but the COVID-19 pandemic might have significantly impacted this process. There was an abrupt transition to online learning and work, severe restrictions on social contact, and uncertainty (Song et al., 2021; Viner et al., 2020). In Spain, for most emerging adults, COVID-19 meant returning to their parents’ home if they had previously moved out, or spending more time with them if they had not (CJE, 2020). That scenario might have limited and altered the opportunities of exploration: education was online; lockdowns severely restricted the current contact with friends/partners and reduced the room for meeting new people; uncertainty about the value of some careers in the “new scenario” impacted the exploration of career paths and, the loss and hard access to jobs during the pandemic reduced the work exploration. Because of this, the times of the COVID-19 pandemic might be considered a critical period, that is, a specific time when significant socio-historical changes are happening (Luo and Hodges, 2022). Given the influence of socioeconomic, cultural, and historical contexts on identity development, this study examines emerging adults from two different cohorts, one pre-pandemic and the other collected during the COVID-19 period, and compares their identity development and psychosocial adjustment.

### Gender differences

Research had identified gender differences in the identity development process (Cramer, 2000; Crocetti et al., 2013, 2015; Marcia, 1966; Schwartz et al., 2011). However, the results of those previous studies were heterogeneous. Cramer (2000), using Marcia’s status model, has reported a higher proportion of men in the identity achievement status group, suggesting that men exhibited greater levels of exploration and commitment. These results are aligned with the findings of Bogaerts et al. (2019), whose research, based on the Dual-Cycle Model, also showed higher levels of commitment and identification with commitment among men. Other studies based on the Dual-Cycle Model have also reported gender differences, finding higher levels of in-depth exploration and ruminative exploration among women (Crocetti et al., 2013; Luyckx et al., 2008b; Merrill et al., 2016), and, unlike the works of Bogaerts, higher commitment scores in women (Crocetti et al., 2015; Johnson and Whisman, 2013; Morsunbul et al., 2014).

Furthermore, the Dual-Cycle Model literature reveals a gap regarding gender differences in the association between identity and adjustment variables (Sánchez‐Queija et al., 2023). In this line, one study found that ruminative exploration had a more negative impact on men’s wellbeing than on women’s, while exploration was positively correlated to the decrease in externalizing problems among women and the increase among men (Ritchie et al., 2013).

Previous studies have also shown that the impact of crises such as natural disasters or pandemics, may vary according to sociodemographic factors as gender (Neumayer and Plümper, 2007; Taylor et al., 2008). Indeed, the COVID-19 pandemic has revealed substantial gender differences in the physical and psychosocial impact on the undergraduate population. A meta-analysis of 27 studies showed that female college students present significantly higher levels of anxiety and depression than male students (Batra et al., 2021). Similarly, a greater proportion of women reported a decline in mental health during the COVID-19 period (Gewalt et al., 2022). Evidence also indicates that undergraduate women experience more pronounced negative effects on academic performance, social isolation, stress, and overall mental health (Prowse et al., 2021). Moreover, gender disparities have been observed in quality of life, general health perception, sleep quality, and loneliness, with women consistently exhibiting less favorable outcomes than their male counterparts (Bermejo-Franco et al., 2022).

Taken together, these findings underscore the importance of examining gender differences in developmental processes across diverse sociocultural contexts, historical periods, and life stages, including emerging adulthood.

### Current study

This study aimed to explore and compare identity development and psychological adjustment among two generations of emerging adults. The study used a cohort design for this purpose: one cohort was surveyed in 2015 (Ch1), and the other in 2020 (Ch2). This study systematically examined gender differences across its aims, which were the following: (1) to examine potential differences in identity scores between the two cohorts, and (2) to compare the association between identity dimensions and psychological adjustment of both generations of emerging adults.

## Method

### Sample

The total sample was comprised of 2,819 college students (38% men and 62% women) from two cohorts. Ch1 was collected in 2015 and had a total of 1,301 emerging adults (40.9% men, 59.1% women; *M* = 20.06, *SD* = 2.03), and Ch2 was collected in 2020 and had a total of 1,518 participants (35.6% men, 64.4% women; *M_age_* = 20.48, *SD* = 2.48). In both cohorts, participants were from the same two Spanish universities, removed for blinded review (Spain), and came from the five main knowledge areas: Arts and Humanities, Social and Legal Science, Science, Health Science, and Engineering and Architecture. The ages of the total sample ranged from 18 to 29 years (*M_age_* = 20.28, *SD* = 2.29). The distribution of both cohorts’ samples, in terms of sociodemographic characteristics, is reported in Table 1.

**Table 1:** Descriptive statistics for sociodemographic variables.

Sociodemographic variables	Ch1	Ch2
University of origin		
University of (blinded for review)	42.6%	50.9%
University of (blinded for review)	57.4%	49.1%
Knowledge field		
Arts and humanities	9.7%	19.4%
Social and legal science	30.5%	19.9%
Science	7.7%	12.8%
Health Science	25%	23%
Engineering and architecture	27.1%	24.9%
Current job status		
Unemployed	81.6%	82.8%
Part-time job	17.6%	14%
Full-time job	0.8%	3.2%
Type of community		
Rural	54.8%	57.6%
Urban	45.2%	42.4%
Perceived family income		
Low	15.6%	15.3%
Medium	70.2%	69.3%
High	14.2%	15.4%
Current living arrangement		
Living at home with parents	66.6%	63.2%
Living with another relative	1.8%	1%
In shared housing or student residence	10.8%	12.8%
Weekdays in shared housing or student residence, weekends with parents	18.3%	19.9%
Living with a partner	1.9%	2.1%
Living with children	—	0.1%
Living alone	0.5%	0.9%

### Instruments

Participants were asked for the following *sociodemographic* var*iables:* gender, age, university, knowledge area, employment status, habitat (rural or urban), current living status, and perceived household income.

*Identity development* was measured with the *Dimensions of Identity Development Scale* (DIDS) (Luyckx et al., 2008a), validated in Spanish by Sánchez‐Queija et al. (2023). This measure has 25 items with a five-point Likert-type scale and five dimensions: *Commitment making* (CM, e.g., ‘Know what I want to do with my future’; α_Ch1_ = 0.91; α_Ch2_ = 0.90), *Identification with commitment* (IC, e.g., ‘Value my plans for the future very much’; α_Ch1_ = 0.90; α_Ch2_ = 0.89), *Exploration in breadth* (EB, e.g., ‘Think about what to do with my life’; α_Ch1_ = 0.86; α_Ch2_ = 0.87), *Exploration in depth* (ED, e.g., ‘Think a lot about the future plans I strive for’; α_Ch1_ = 0.79; α_Ch2_ = 0.80) and *Ruminative exploration* (RE, e.g., ‘Worry about what I want to do with my future’; α_Ch1_ = 0.85; α_Ch2_ = 0.87). Metric and scalar invariance between cohorts were confirmed (see Supplementary material).

*Psychological adjustment* was measured using two variables:

*Wellbeing* was measured with the Spanish version (De la Fuente et al., 2017) of the *Flourishing Scale* (Diener et al., 2010). The scale has eight items with a seven-point Likert-type scale (e.g., ‘I lead a purposeful and meaningful life’). Scale reliability was α_Ch1_ = 0.81 and in α_Ch2_ = 0.82. Metric and Scalar invariance between cohorts were confirmed (see Supplementary material).

*Psychological distress* was measured with the Spanish version (Bados et al., 2005) of the *Depression Anxiety Stress Scales* (DASS-21) (Lovibond and Lovibond, 1995). The scale has 21 items with a four-point Likert-type scale (e.g., ‘I am a good person and live a good life’) that measures anxiety, depression, and stress symptoms. The global scale score is an indicator of psychological distress. Reliability of this scale was α_Ch1_ = 0.89 and in α_Ch2_ = 0.93. Metric and Scalar invariance between cohorts were confirmed (see Supplementary material).

### Procedure

The research staff of both cohorts’ data collection contacted the faculties of the University of (removed for blinded review) to explain the study’s purpose and request the participation of professors and students. Students were informed about the study aims and their participation, which was voluntary and anonymous. Questionnaires were administered to Ch1 in paper-based format during class hour. Ch2 was surveyed 2 months after the implementation of preventive measures—including the closure of non-essential services, local lockdowns, social distancing, and mandatory mask use. Consequently, data for Ch2 were collected using online questionnaires whose link was facilitated to the students by the professors and the research team. Both data collections and this study were approved by the Ethics Committee of (removed for blinded review).

### Analysis plan

Descriptive analyses were performed to explore the five dimensions of identity development scores of Ch1 and Ch2. Multivariate Generalized Linear Model (GLM) analyses were also performed to explore potential differences between the identity dimension scores of Ch1 and Ch2 men and women. To this end, the interaction between gender and cohort was included in the GLM analyses. Effect sizes for GLM analyses (*η_p_^2^*) were verified with Cohen’s (2013) metrics. The GLM analyses were conducted in SPSS v.29. Subsequently, preliminary correlation analyses were conducted to analyze the relationship between the identity dimensions and the psychological adjustment’s variables. Finally, moderation analyses were performed to explore whether the cohort and gender moderated the relationship between identity and psychological adjustment. The age was controlled in all analyses. Said moderation analyses were conducted with the PROCESS macro (Hayes, 2022) in the SPSS v29 program, which uses hierarchical regression analyses as the base for the exploration of the moderation effects. The variables and the interaction terms used to explore the moderation effects were introduced in the models following the hierarchical principle of regression modeling. Effect sizes for moderation analyses (*R^2^*) were verified with Cohen’s (1988) metrics. Metric and scalar invariance were conducted with MPlus 8.9 (see Supplementary material).

## Results

To tackle the first objective, descriptive and GLM analyses were conducted to examine potential differences in the scores of identity dimension variables between both cohorts. The results of descriptive and GLM analyses are shown in Table 2. On the one hand, Ch1 participants exhibited significantly higher scores in commitment making (small size effect), identification with commitment (small size effect), and exploration in depth (negligible size effect) than Ch2 participants. On the other hand, Ch2 participants showed higher scores for ruminative exploration (negligible size effect). The interaction between gender and cohort was only significant for one of the dependent variables, exploration in depth, but with a negligible effect size, *F*(1, 2,782) = 4.09, *p* = 0.043, *η_p_^2^* = 0.001.

**Table 2:** Descriptive statistics and GLM results of the comparison of identity dimensions between Ch1 and Ch2.

	Women		Men		Total		*SS*	df	*F*	*p*	*η_p_^2^*
	Ch1	Ch2	Ch1	Ch2	Ch1	Ch2					
	M (SD)	M (SD)	M (SD)	M (SD)	M (SD)	M (SD)					
Identity dimensions											
Commitment making	3.63 (0.90)	3.40 (0.97)	3.70 (0.88)	3.58 (0.96)	3.66 (0.89)	3.47 (0.97)	21.67	1, 2,782	25.00	<0.001	0.009
Identification with commitment	3.58 (0.87)	3.29 (0.99)	3.68 (0.87)	3.54 (0.96)	3.62 (0.87)	3.38 (0.99)	30.28		35.04	<0.001	0.012
Exploration in breadth	3.85 (0.80)	3.85 (0.87)	3.78 (0.80)	3.87 (0.84)	3.82 (0.80)	3.86 (0.86)	0.64		0.93	0.335	0.000
Exploration in depth	3.48 (0.80)	3.33 (0.86)	3.37 (0.78)	3.37 (0.87)	3.44 (0.80)	3.35 (0.86)	6.39		9.39	0.002	0.003
Ruminative exploration	3.19 (0.96)	3.38 (1.04)	3.09 (1.00)	3.24 (1.05)	3.15 (0.98)	3.33 (1.05)	17.67		17.18	<0.001	0.006
Flourishing	46.87 (5.09)	45.27 (6.05)	45.64 (6.11)	44.69 (6.79)	46.37 (5.55)	45.06 (6.32)	992.41		27.86	<0.001	0.010
General distress	33.47 (21.47)	44.10 (27.51)	29.26 (20.98)	36.34 (25.70)	31.76 (21.37)	41.35 (27.13)	48894.16		81.67	<0.001	0.029

The effect sizes for *η*_p_^2^ are: small (0.01), medium (0.06), or large (0.14) effect sizes.

To address the second objective, preliminary correlation analyses were performed. Correlations shown in Table 3 were performed to explore the associations between identity and psychological adjustment variables in both cohorts for men and women separately.

**Table 3:** Correlations between identity and adjustment variables in Ch1 and Ch2 men and women.

Women	Cohort 1					Cohort 2				
	CM	IC	EB	ED	RE	CM	IC	EB	ED	RE
Wellbeing	0.36***	0.39***	0.11**	0.17***	−0.22***	0.44***	0.47***	0.07*	0.16***	−0.32***
Distress	−0.15***	−0.16***	0.16***	0.11**	0.22***	−0.18***	−0.26***	0.16***	0.08*	0.29***

Men	Cohort 1					Cohort 2				
	CM	IC	EB	ED	RE	CM	IC	EB	ED	RE
Wellbeing	0.48***	0.51***	0.04	0.13**	−0.33***	0.49***	0.55***	0.11*	0.25***	−0.33***
Distress	−0.30***	−0.27***	0.22***	0.13**	0.36***	−0.24***	−0.31***	0.16***	0.10*	0.34***

Total	Cohort 1					Cohort 2				
	CM	IC	EB	ED	RE	CM	IC	EB	ED	RE
Wellbeing	0.40***	0.43***	0.08**	0.16***	−0.26***	0.45***	0.49***	0.08***	0.20***	−0.32***
Distress	−0.22***	−0.21***	0.19***	0.13***	0.28***	−0.21***	−0.29***	0.16***	0.09***	0.31***

*p < 0.05, **p < 0.01, ***p < 0.001.

As shown in Table 3, wellbeing is positively associated with both commitment dimensions—commitment making and identification with commitment—as well as with exploration in breadth and exploration in depth. In contrast, wellbeing is negatively associated with ruminative exploration. Conversely, psychological distress is negatively associated with the two commitment dimensions and positively associated with exploration in breadth, exploration in depth, and ruminative exploration. The associations between identity dimensions and psychological adjustment variables are virtually the same between gender and cohorts, with the exception that the correlation between wellbeing and exploration in breadth was significant in Ch1 women and Ch2 men and women, but not in Ch1 men.

Moderation analyses were also conducted to fully address objective 2, that is, to examine the role of cohort and gender among emerging adults on the relationship between identity dimensions on psychological adjustment variables. Results are shown in Table 4.

**Table 4:** Moderation effects of cohort and gender on the association between identity and adjustment.

	Wellbeing				Psychological distress			
	*β* (*p*)	95% CI (LLCI, ULCI)	*t*	*R* ^2^	*β* (*p*)	95% CI (LLCI, ULCI)	*t*	*R* ^2^
Models 1 and 2								
CM	3.12 (>0.001)***	(2.69, 3.54)	14.46	0.21***	−6.28 (>0.001)***	(−8.17, −4.39)	−6.51	0.09***
Cohort	−2.64 (0.001)**	(−4.24, −1.04)	−3.23		10.70 (0.003)**	(3.53, 17.86)	2.93	
Gender	4.59 (>0.001)***	(2.96, 6.23)	5.51		−1.67 (0.654)	(−8.99, 5.65)	−0.45	
CM*Cohort	0.51 (0.021)*	(0.08, 0.94)	2.31		−0.71 (0.473)	(−2.64, 1.23)	−0.72	
CM*Gender	−0.94 (>0.001)***	(−1.38, −0.50)	−4.20		1.99 (0.048)*	(0.02, 3.96)	1.98	
Models 3 and 4								
IC	3.41 (>0.001)***	(3.00, 3.83)	16.09	0.25***	−5.81 (>0.001)***	(−7.70, −3.93)	−6.06	0.11***
Cohort	−2.44 (0.002)**	(−3.99, −0.88)	−3.08		17.57 (>0.001)***	(10.53, 24.60)	4.90	
Gender	5.11 (>0.001)***	(3.53, 6.69)	6.35		−0.58 (0.874)	(−7.72, 6.57)	−0.16	
IC*Cohort	0.51 (0.020)*	(0.08, 0.93)	2.33		−2.80 (0.004)**	(−4.73, −0.87)	−2.85	
IC*Gender	−1.05 (>0.001)***	(−1.48, −0.62)	−4.78		1.56 (0.117)	(−0.39, 3.51)	1.57	
Models 5 and 6								
EB	0.59 (0.029)*	(0.06, 1.11)	2.18	0.02***	5.01 (>0.001)***	(2.89, 7.13)	4.64	0.08***
Cohort	−1.60 (0.137)	(−3.71, 0.51)	−1.49		7.85 (0.072)	(−0.70, 16.41)	1.80	
Gender	1.03 (0.351)	(−1.13, 3.19)	0.93		7.33 (0.101)	(−1.43, 16.09)	1.64	
EB*Cohort	0.07 (0.811)	(−0.47, 0.60)	0.24		0.31 (0.778)	(−1.87, 2.49)	0.28	
EB*Gender	−0.05 (0.859)	(−0.60, 0.50)	−0.18		−0.33 (0.769)	(−2.56, 1.90)	−0.29	
Models 7 and 8								
ED	1.36 (>0.001)***	(0.83, 1.88)	5.09	0.05***	3.38 (0.002)**	(1.22, 5.55)	3.06	0.06***
Cohort	−2.30 (0.016)*	(−4.17, −0.44)	−2.42		10.52 (0.008)**	(2.79, 18.26)	2.67	
Gender	2.16 (0.025)*	(0.27, 4.05)	2.24		7.78 (0.052)	(−0.06, 15.63)	1.95	
ED*Cohort	0.33 (0.229)	(−0.21, 0.86)	1.20		−0.30 (0.787)	(−2.52, 1.91)	−0.27	
ED*Gender	−0.40 (0.147)	(−0.94, 0.14)	−1.45		−0.51 (0.656)	(−2.76, 1.74)	−0.45	
Models 9 and 10								
RE	−1.79 (>0.001)***	(−2.19, −1.39)	−8.71	0.11***	6.80 (>0.001)***	(5.14, 8.45)	8.05	0.13***
Cohort	0.50 (0.491)	(−0.93, 1.93)	0.69		1.55 (0.604)	(−4.32, 7.42)	0.52	
Gender	−0.47 (0.521)	(−1.92, 0.97)	−0.64		10.23 (>0.001)***	(4.29, 16.17)	3.38	
RE*Cohort	−0.48 (0.027)*	(−0.90, −0.05)	−2.21		2.00 (0.024)*	(0.26, 3.73)	2.26	
RE*Gender	0.48 (0.028)*	(0.05, 0.91)	2.20		−1.54 (0.087)	(−3.30, 0.22)	−1.71	

*p < 0.05, **p < 0.01, ***p < 0.001. CM, commitment making; IC, identification with commitment; EB, exploration in breadth; ED, exploration in depth, RE, ruminative exploration. No statistically significant interaction effects were found between identity dimensions, cohort, and gender. The men and Ch1 were the reference groups for the comparisons. The effect sizes for *R*^2^ are: small (0.01), medium (0.09), or large (0.25) effect sizes.

**Figure 1:**
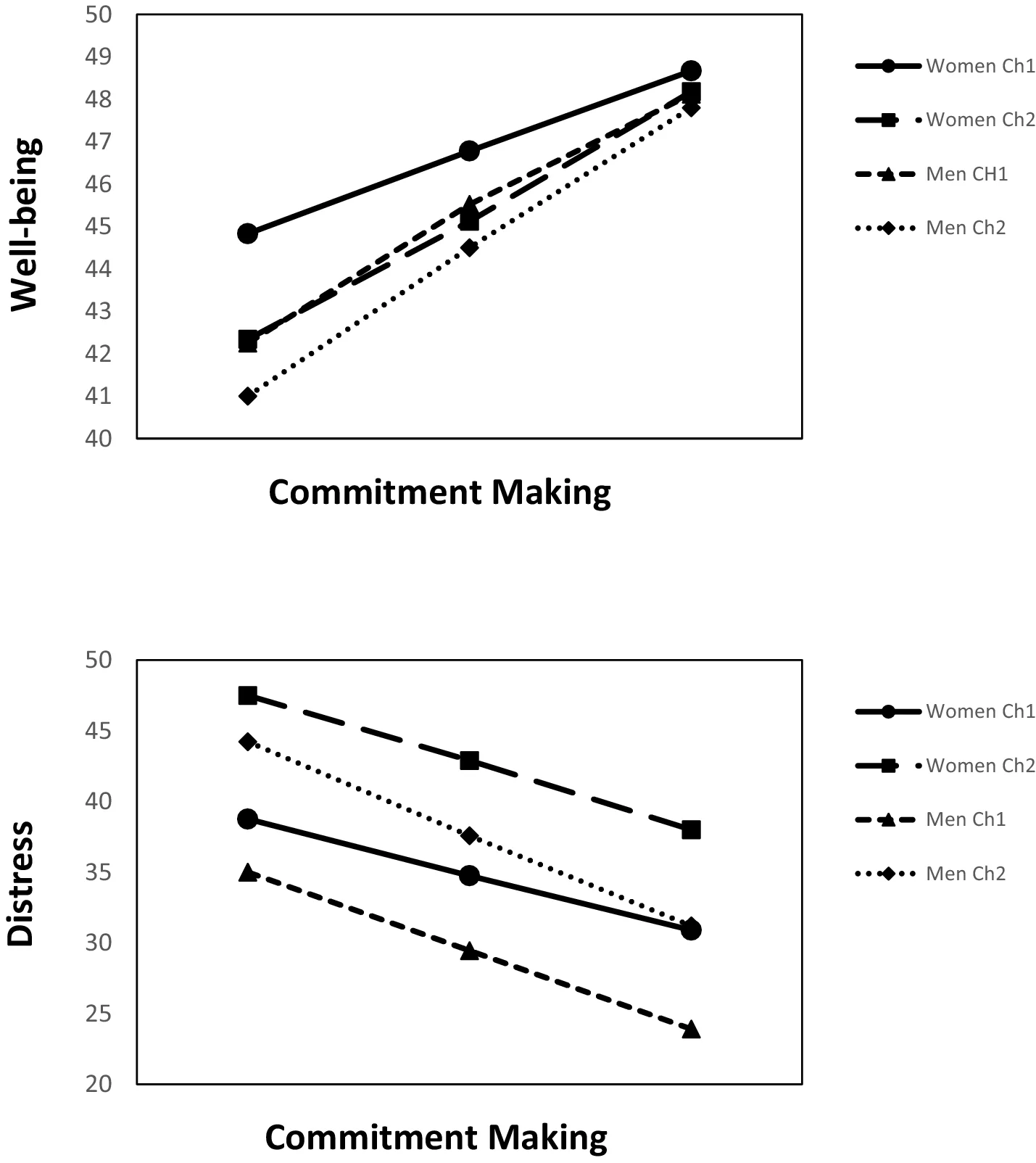
Moderation of cohort and gender on the association between commitment making and well-being (upper), and moderation of gender on the association between commitment making and psychological distress (lower).

**Figure 2:**
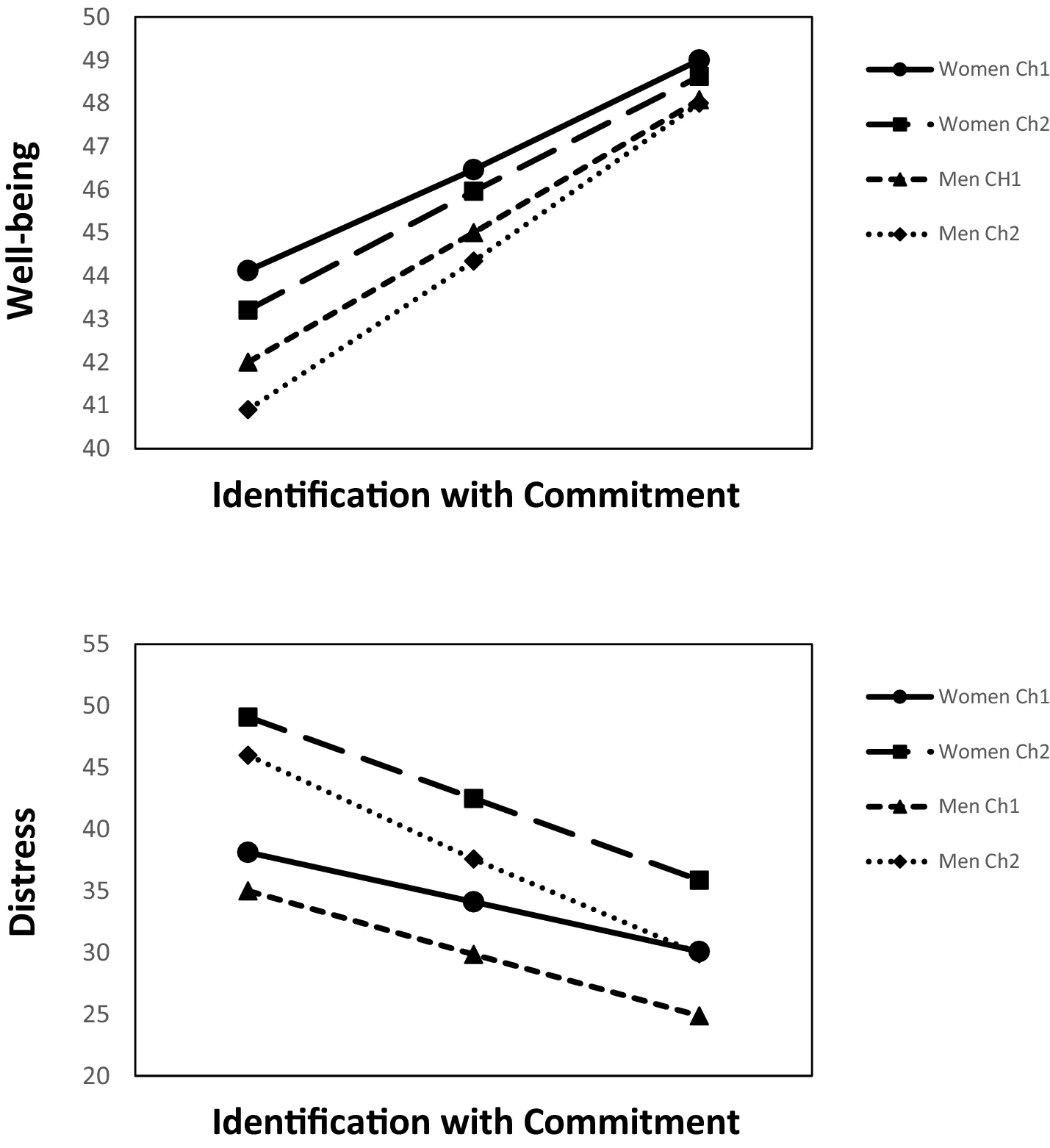
Moderation of cohort and gender on the association between identification with commitment and well-being (upper), and moderation of cohort on the association between identification with commitment and psychological distress (lower).

**Figure 3:**
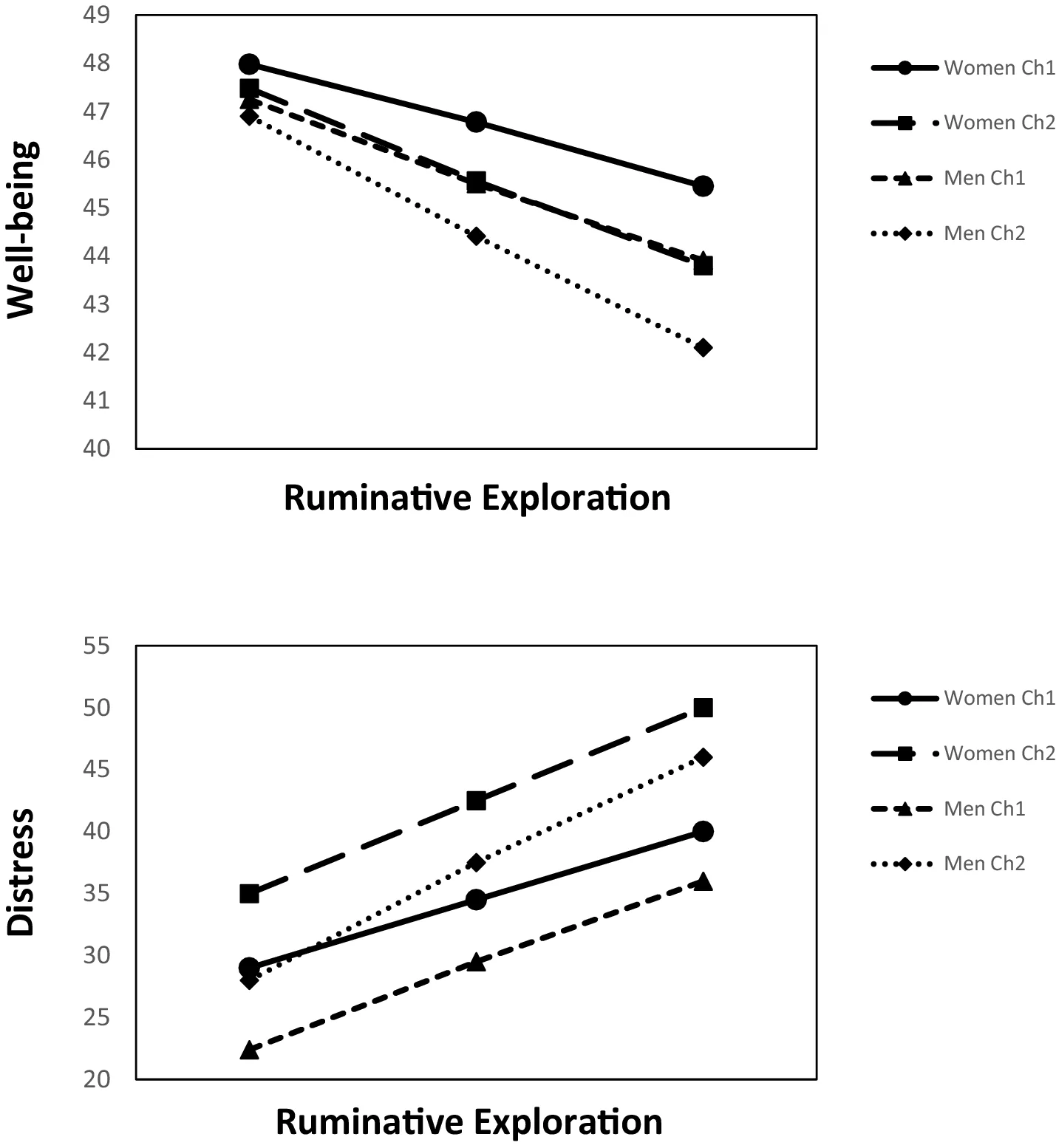
Moderation of cohort and gender on the association between ruminative exploration and well-being (upper), and moderation of cohort on the association between ruminative exploration and psychological distress (lower).

Table 4 shows that the cohort significantly moderated the relationship between: (a) commitment making and wellbeing, (b) identification with commitment and wellbeing, (c) identification with commitment and psychological distress, (d) ruminative exploration and wellbeing, and (e) ruminative exploration and psychological distress. Gender also moderated the relationship between: (a) commitment making and wellbeing, (b) commitment making and psychological distress, (c) identification with commitment and wellbeing, and (d) ruminative exploration and wellbeing. Figures 1–3 show those moderation effects in further detail. All associations between identity dimensions and psychological adjustment variables significantly moderated by cohort were stronger in Ch2 participants and in men.

## Discussion

This study aimed to explore and compare identity development and psychological adjustment across two generations of emerging adults. The Dual-Cycle Model of identity was selected to explore and measure identity dimensions, and a cohort design (one collected in 2015, Ch1, and another collected in 2020, during the pandemic period, Ch2) was used to tackle the study’s main goal. The two specific aims of this study were: (1) to examine potential differences in identity scores between Ch1 and Ch2; and (2) to compare the associations between identity dimensions and psychological adjustment across both generations of emerging adults. Gender differences were also explored in both objectives.

### Identity development and psychological adjustment of two different generations

In Erikson’s and Marcia’s earliest formulations, identity development was conceptualized as a psychosocial construct. Nowadays, the identity is understood as a form of self-development whose trajectory is shaped by the sociohistorical context in which individuals construct their sense of self (Galliher et al., 2017). While cross-national and cross-cultural variations in identity development have previously been documented (e.g., Low et al., 2005), to the best of our knowledge, little research has investigated the ways in which historical circumstances may shape identity construction within the same society and culture using comparable samples. The findings of this study suggest that the identity development process—and its association with psychological adjustment during emerging adulthood—is related to the generational context to which these young people belong. It is also important to note that Ch2’s data was collected during the COVID-19 pandemic, which makes plausible the idea that the COVID-19 period interfered with the self-development process of the identity and the psychosocial adjustment. This study does not have enough evidence to determine whether this potential impact comes from the period, the generation of each cohort, or an interplay of both, but this highlights the need to develop further research studies in that direction. Another important consideration before proceeding to the discussion concerns the effect sizes of the results. Most effect sizes of the analyses conducted had small-medium indexes. Although most do not display large effects, some authors state that modest effect sizes are often acceptable in social sciences, such as psychology, particularly when analyzing complex human behavior. For example, according to another author Ozili (2023), “a low *R*^2^ of at least 0.1 (or 10 percent) is acceptable on the condition that some or most of the predictors or explanatory variables are statistically significant”. In result, most of the findings have been considered as valid observations and interpreted in consequence.

The levels of exploration in breadth, in depth, and ruminative were similar in both cohorts, with only minimal differences that did not reach a significant effect size. This finding highlight that the exploration processes might operate similarly in the different socioeconomic and historical events’ constraints faced by Ch1 and Ch2. In contrast, Ch2 participants, comprising both men and women, reported significantly lower scores in commitment making and identification with commitment compared to Ch1. That is, participants in Cohort 2 are less certain about the path they want to follow in life or what they want to do in the future, and the plans they do have provide them with less self-confidence and a weaker sense of security. Notably, both cohorts have experienced social, educational, and economic uncertainty stemming from the global financial crisis of the late 2000s (Mieszala, 2019). That socioeconomic crisis has persisted in the ensuing decades in Spain (Espinosa et al., 2018), manifesting through a high rate of youth unemployment, prevalence of temporary contracts, and a prolonged average age of leaving the parental home—29.4 years for women and 30.5 years for men (Eurostat, 2025a, 2025b, 2025c). Previous research has shown that emerging adults encounter significant challenges in establishing and maintaining commitments when operating within contexts characterised by limited and precarious opportunities (Skhirtladze et al., 2021). Such constraints in the social and economic spheres may result in: (a) fewer potential commitments available to young people, thereby hindering the commitment making process, and (b) a higher likelihood of commitments being selected out of necessity or availability rather than personal desirability, which can weaken identification with commitment when those choices diverge from individual values, expectations, or goals.

Both Ch1 and Ch2 may have experienced the aforementioned difficulties, although these challenges were likely intensified for Ch2 due to the disruptions associated with the COVID-19 pandemic and its aftermath (Hiscott et al., 2020; Song et al., 2021). The COVID-19 pandemic significantly altered the college learning methodology and context, leading to the transition from learning in person to an online learning methodology, and reducing the ratio of students per class (Viner et al., 2020). The pandemic also limited physical and social contact with relatives, partners, friends, and classmates for safety concerns, increased difficulty in maintaining current relationships and jobs, reduced social, educational and work opportunities, caused the loss of close ones because of the disease, and led to sadness, fear or anger due to the pandemic circumstances and its consequences (Torales et al., 2020). Those who already had commitments during the COVID-19 pandemic might also have faced difficulties in maintaining their current commitments, such as keeping a job in times of economic uncertainty or friendships/romantic relationships during times of restricted social contact (Estlein et al., 2022). The dystopic conditions derived by lockdowns and other restrictions could have led to a life crisis and deliberated -or forced-introspection of current life conditions and commitments (Żurko and Słowińska, 2022). Hypothetically speaking, these struggles and reassessments might have weakened their identification with commitments if those commitments were perceived as less sustainable or appealing. At the same time, those who were trying to establish new commitments might have faced significant restraints due to the limitations of the pandemic, which could have reduced the likelihood of choosing their target commitments. That scenario created a more restricted developmental context for Ch2 emerging adults than the experienced by Ch1, far removed from what emerging adulthood is usually considered: a stage of possibilities, open paths, and free exploration.

The results of the moderation analysis also highlight two points: (a) commitment making, identification with commitment, and ruminative exploration were the dimensions with stronger associations with psychological adjustment in both cohorts, and (b) the association between those dimensions and adjustment was stronger in Ch2 participants.

Identity research emphasizes the association of commitment processes with adjustment and mental health during emerging adulthood (Luyckx et al., 2008a, 2013b; Schwartz et al., 2011, 2012, 2015). Indeed, our study’s findings underline a significant association of the commitment processes with the psychological adjustment of emerging adults. Despite prior research supporting the existence of this association during adolescence (De Lise et al., 2024), other studies have found that this association is stronger in the emerging adulthood stage (Luyckx et al., 2013a), underscoring the central role these processes play in emerging adults’ self-definition (Waterman and Archer, 1990). The exploration in breadth and in depth, on the other hand, had modest associations with the adjustment. Both exploration dimensions might have a stronger association with the adjustment during adolescence (Luyckx et al., 2013a), but their role in emerging adults’ adjustment might be secondary to the commitment processes. However, our findings show that ruminative exploration, the third type of exploration, might be related to the emerging adults’ adjustment as solidly as the commitment processes. It is possible that ruminative exploration could interfere with commitment formation and identification processes and, directly or indirectly, be associated with negative adjustment outcomes, as our results have shown in both cohorts.

This study also highlights a stronger association between identity processes and adjustment outcomes in Ch2 participants. Identity development provides a sense of continuity over time while navigating life challenges (Erikson, 1968), a framework that guides decisions on career, relationships, and values (Marcia, 1980), and resilience in adapting to psychosocial and economic challenges (Kroger, 2006). During the pandemic, however, emerging adults faced a range of additional pressures and challenges that disrupted multiple areas of life and socio-economic stability (Torales et al., 2020). In times of stability, factors such as physical health, financial security, access to education and work, social contact, and family functioning are critical to adjustment (Aoun et al., 2004; Lee et al., 2022). However, in times of crisis and uncertainty, access to education, employment, financial stability, and social connections becomes more precarious, unstable, and fragile. In that scenario, it is feasible that having a clear sense of self—shaped by the establishment of commitments and their identification with one’s values and goals—may be related to fewer mental health issues often triggered by crises. Identity processes, therefore, may be more strongly associated with wellbeing and psychological distress in times of adversity as the COVID-19 pandemic, thereby strengthening the identity-adjustment association during this period.

### Gender differences in the identity development and psychological adjustment

Gender emerged as a significant moderator in the relationship between identity dimensions and adjustment in both cohorts. Men exhibited stronger associations between identity dimensions and adjustment variables compared to women, supporting that these associations might be influenced by gender. Specifically, men in both cohorts revealed stronger associations between commitment, identification with commitment, ruminative exploration, and both wellbeing and psychological distress. Despite similar results having been found in a previous study (Ritchie et al., 2013), other authors have not found significant gender differences in this association (Berzonsky, 2011; Luyckx et al., 2013b), which may point to a complex interplay between identity, adjustment, gender, and, probably, the sociocultural context of the participants.

Societal expectations about gender roles may partially account for the findings, even in societies that might be considered high in gender equality (Saewyc, 2017). On the one hand, contemporary societies continue to emphasize taking action and attaining goals in men while promoting empathy and caregiving in women (Matud et al., 2019), that is, men are expected to show more agentic characteristics, while women are expected to be more communal (Kosakowska-Berezecka et al., 2023; Moskowitz et al., 1994). Agency is more closely associated with a focus on self—and, therefore, identity development—than communion, which is strongly linked to a focus on others (Helgeson, 1994). On the other hand, emerging adults who face life challenges with a strong sense of agency tend to attain greater progress in the exploration and consolidation of their identity (Booker et al., 2021; Schwartz et al., 2005). This means that, although agency and identity resolution are associated with wellbeing in both men and women, it is not surprising that this association is stronger among those who align more closely with the socially established stereotype of focusing on themselves, seeking self-definition, and consolidating their identity: men. This might account for the findings of this study, that is, why associations between identity processes and adjustment variables could be stronger in men of both cohorts. Future research lines would benefit from the use of a gender perspective in the study of identity to identify the causes that underlie in those differences.

### Limitations and future research

One of the main limitations of this study is related to the composition of both cohorts’ samples. The emerging adulthood stage comprises youths between age 18 and 29 from heterogeneous backgrounds: college students, vocational training students, part-time or full-time workers, unemployed youths who neither work nor study, homemakers, married youths, parents… Moreover, the early and late emerging adulthood years may involve different challenges and demands for young people. However, this study only analyzed two samples of college emerging adults whose mean age was 20, given that most participants were between 18 and 23 years old. Having a sample composed exclusively of university students and with a mean age of 20 may limit to some extent the generalizability of the results to other emerging adults from different backgrounds. Nevertheless, the findings have been considered as valid observations and interpreted in consequence.

To minimize the potential impact of age, this variable was statistically controlled in all analyses conducted in this study. Despite its limitations, this study offers valuable insights into the complex interplay between generational cohorts, identity development, and psychological adjustment during emerging adulthood. The unique circumstance that the second cohort was assessed during the unprecedented social crisis triggered by the COVID-19 pandemic—within a country that implemented particularly stringent public health measures—enabled the study to operate as a natural observation, one that will be difficult to replicate in future research.

Another limitation of this study lies in the different methods used for the data collection of each cohort (paper administration in Ch1 and online survey administration in Ch2). The authors are aware that both methods could lead to discrepancies and biases in the answers of the participants, such as social desirability, different levels of self-disclosure, and differences in the reporting of negative variables like psychological distress or ruminative exploration questions. The decision to change the method of data collection in Ch2 was a direct consequence of the conditions of the COVID-19 pandemic: social and mobility restrictions derived from the lockdowns. To reduce the bias to a minimum, the research team: organized the items and scales of the questionnaire of Ch2 in a similar order, followed the same method to contact participants (survey them during the college classes in a space in hour provided by the professor in charge), offered contact and availability to the participants to answer any doubts they had during the completion, and guaranteed the anonymity of the participants. However, the team will include more control measures to ensure that future data collections address the biases posed by different collection methods.

The study confirmed that commitment making, identification with commitment, and ruminative exploration might have a higher association with adjustment than exploration in breadth and in depth processes. Previous research has already pointed out how the establishment of commitments and identification with them might promote maturation, increase wellbeing, and protect against mental health challenges, while ruminative exploration could negatively interfere with this process and indirectly reduce wellbeing while heightening mental health symptoms. This study supports that line of research from an observational and exploratory perspective.

The disparities between Ch1 and Ch2 participants also highlight that identity development might have a higher association with adjustment outcomes during times of crisis—such as the pandemic. This developmental process has functions such as adaptation, developing coping mechanisms, guiding the decision-making process, and promoting resilience to face the challenges of psychosocial and economic circumstances, which might be related to better mental health and wellbeing during times of crisis. In other words, identity could be “a pillar to cling to in times of crisis”.

The observed gender differences also highlight that the association between identity development and adjustment might be stronger in men. They also reflect the need to study the underlying mechanisms of the identity development gender differences to develop specific interventions that address the distinct challenges faced by men and women. It is crucial to overcome those potential disparities and promote an adequate process that fosters good identity development and adjustment in the mid and long term. Moreover, the recognition of gender disparities in identity development and adjustment highlights the need to adopt a gender perspective in research and the development of intervention programs.

Pandemics and health crises are complex phenomena, and several factors can lead to their emergence, such as the spread of new viruses, the climate crisis, social conditions, and global interactions. Moreover, their impact on the emerging adults’ lives can be tangled to other generational effects derived from diverse socioeconomic and cultural changes, which creates a complex scenario that demands quality research and the rigorous control of generational effects under study. Studies like the one we report are necessary to help us understand how the developmental processes might operate during crisis times like the COVID-19 pandemic. While it is crucial to develop early warning systems, vaccines, or international protocols to prevent the spread of diseases, it is equally important to understand how development was impacted and design tools to counteract their effects if a similar situation were to occur in the future.

## Data Availability

The data that support the findings of this study are available on https://doi.org/10.12795/11441/175124.
