# Correction: The importance of the socio-historical context: similarities and differences in identity development and psychological adjustment across two cohorts of Spanish college emerging adults

**DOI:** 10.3389/fpsyg.2026.1952205

**Published:** 2026-08-27

**Authors:** Paula Domínguez-Alarcón, Inmaculada Sánchez-Queija, Agueda Parra

**Affiliations:** Department of Developmental and Educational Psychology, Universidad de Sevilla, Sevilla, Spain

**Keywords:** emerging adulthood, gender differences, identity, mental health, wellbeing

This funder paragraph was erroneously included:

The author(s) declared that financial support was received for this work and/or its publication. This work was supported by five grants issued by: (a) the Ministerio de Economía y Competitividad, Agencia Estatal de Investigación (Spanish Ministry of Economy and Competitiveness, EDU2013-45687-R), (b) the Ministerio de Ciencia, Innovación y Universidades, Agencia Estatal de Investigación (Spanish Ministry of Science, Innovation and Universities) and the European Regional Development Fund (RTI2018-097405-B-I00), (c) the Consejería de Transformación Económica, Industria, Conocimiento y Universidades de la Junta de Andalucía (Regional Ministry of Economic Transformation, Industry, Knowledge and Universities of the Regional Government of Andalucía, PROYEXCEL_00766), (d) the Project PID2024-155825NB-I00, funded by MICIU/AEI/10.13039/501100011033 and by the ERDF, EU, and (e) the Conserjería de Transformación Económica, Industria, Conocimiento y Universidades—Junta de Andalucía (Ministry of Economic Transformation, Industry, Knowledge and Universities of the regional government of Junta de Andalucía—predoctoral contract. Beneficiary: Paula Domínguez Alarcón, PREDOC_01326).

The correct **Funding** statement appears below:

The author(s) declared that financial support was received for this work and/or its publication.

-Project PID2024-155825NB-I00, funded by MICIU/AEI/10.13039/501100011033 and by ERDF, EU.

-Conserjería de Transformación Económica, Industria, Conocimiento y Universidades, Junta de Andalucía (Beneficiary: Paula Domínguez Alarcón, PREDOC_01326).

The original version of this article has been updated.

