# Supplementary material for: The importance of the socio-historical context: similarities and differences in identity development and psychological adjustment across two cohorts of Spanish college emerging adults
Source: Front Psychol. 2026 May 28;17:1739488. doi: 10.3389/fpsyg.2026.1739488 (PMC13253958; doi:10.3389/fpsyg.2026.1739488)
Supplement: Supplementary file 1 [file Data_Sheet_1.DOCX]

**Supplementary Material**

The assumption of metric and scalar invariance of all (sub)scales across cohorts was tested to validate all the analyses. Multi-group confirmatory factor analyses were conducted to check three invariance indices: the difference in chi-square value (Δχ^2^), the CFI difference value (ΔCFI), and the RMSEA difference value (ΔRMSEA) for metric invariance (configural model vs metric model) and scalar invariance (metric model vs scalar model). It would be necessary to meet two or more of the following criteria to reject the assumption of invariance: Δχ^2^ significant at *p* < .05, ΔCFI ≥ .01, and/or ΔRMSEA ≥ .01. Invariance tests were conducted in MPlus v.8.9.

1. **Identity**

Two changes were conducted to improve the model fit: 1) Exclude item 7 (e.g., “Future plans give me self-confidence”) of the *identification with commitment* subscale from the analysis due to its absence in the cohort 2 dataset; 2) To improve the model fit of *exploration in breadth,* items 11 (e.g, “Think about the direction I want to take in my life”) and 12 (e.g., “Think a lot about how I see my future”) were correlated in both cohorts.

***Commitment Making***

| **Modelo** | **χ² (df)** | **CFI** | **RMSEA** | **Invariance** | **Δχ^2^ (Δdf)** | ***p*** | **ΔCFI** | **ΔRMSEA** |
| --- | --- | --- | --- | --- | --- | --- | --- | --- |
| Configural | 52.32 (10) | .993 | .048 |  |  |  |  |  |
| Metric | 62.01 (14) | .993 | .043 | Metric | 8.29 (4) | .082 | 0 | .005 |
| Scalar | 71.81 (18) | .992 | .040 | Scalar | 6.30 (4) | .178 | .001 | .003 |

***Identification with Commitment***

| **Modelo** | **χ² (df)** | **CFI** | **RMSEA** | **Invariance** | **Δχ^2^ (Δdf)** | ***p*** | **ΔCFI** | **ΔRMSEA** |
| --- | --- | --- | --- | --- | --- | --- | --- | --- |
| Configural | 148.75 (4) | .966 | .139 |  |  |  |  |  |
| Metric | 162.68 (7) | .963 | .106 | Metric | 2.96 (3) | .399 | .003 | .03 |
| Scalar | 200.76 (10) | .955 | .101 | Scalar | 27.90 (3) | <.001 | .008 | .008 |

***Exploration in Breadth***

| **Modelo** | **χ² (df)** | **CFI** | **RMSEA** | **Invariance** | **Δχ^2^ (Δdf)** | ***p*** | **ΔCFI** | **ΔRMSEA** |
| --- | --- | --- | --- | --- | --- | --- | --- | --- |
| Configural | 161.23 (8) | .968 | .101 |  |  |  |  |  |
| Metric | 175.05 (12) | .966 | .085 | Metric | 3.33 (4) | .504 | .002 | .016 |
| Scalar | 199 (16) | .962 | .078 | Scalar | 10.86 (4) | .028 | .004 | .007 |

***Exploration in Depth***

| **Modelo** | **χ² (df)** | **CFI** | **RMSEA** | **Invariance** | **Δχ^2^ (Δdf)** | ***p*** | **ΔCFI** | **ΔRMSEA** |
| --- | --- | --- | --- | --- | --- | --- | --- | --- |
| Configural | 399.15 (10) | 898 | .145 |  |  |  |  |  |
| Metric | 424.23 (14) | .89 | .126 | Metric | 2.17 (4) | .705 | .008 | .019 |
| Scalar | 489.52 (18) | .88 | .119 | Scalar | 51.26 (4) | >.001 | .01 | .007 |

***Ruminative Exploration***

| **Modelo** | **χ² (df)** | **CFI** | **RMSEA** | **Invariance** | **Δχ^2^ (Δdf)** | ***p*** | **ΔCFI** | **ΔRMSEA** |
| --- | --- | --- | --- | --- | --- | --- | --- | --- |
| Configural | 279.02 (10) | .951 | .12 |  |  |  |  |  |
| Metric | 315.61 (14) | .945 | .11 | Metric | 20.63 (4) | >.001 | .006 | .01 |
| Scalar | 354.24 (18) | .939 | .10 | Scalar | 25.86 (4) | >.001 | .006 | .01 |

1. **Well-being**

| **Modelo** | **χ² (df)** | **CFI** | **RMSEA** | **Invariance** | **Δχ^2^ (Δdf)** | ***p*** | **ΔCFI** | **ΔRMSEA** |
| --- | --- | --- | --- | --- | --- | --- | --- | --- |
| Configural | 410.52 (40) | .929 | .07 |  |  |  |  |  |
| Metric | 455.71 (47) | .92 | .068 | Metric | 64.68 (7) | >.001 | .009 | .002 |
| Scalar | 524.73 (54) | .91 | .068 | Scalar | 60.84 (7) | >.001 | 0 | .01 |

1. **Psychological Distress**

| **Modelo** | **χ² (df)** | **CFI** | **RMSEA** | **Invariance** | **Δχ^2^ (Δdf)** | ***p*** | **ΔCFI** | **ΔRMSEA** |
| --- | --- | --- | --- | --- | --- | --- | --- | --- |
| Configural | 3878.35 (340) | .849 | .075 |  |  |  |  |  |
| Metric | 3987.99 (359) | .84 | .074 | Metric | 137.74 (19) | >.001 | .009 | .001 |
| Scalar | 4289.18 (378) | .83 | .075 | Scalar | 325.15 (19) | >.001 | .01 | .001 |
